# New Methods, New Concepts: What Can Be Applied to Freshwater Periphyton?

**DOI:** 10.3389/fmicb.2020.01275

**Published:** 2020-06-23

**Authors:** Yulia I. Gubelit, Hans-Peter Grossart

**Affiliations:** ^1^Laboratory of Freshwater Hydrobiology, Zoological Institute, Russian Academy of Science, Saint Petersburg, Russia; ^2^Institute for Biochemistry and Biology, University of Potsdam, Potsdam, Germany; ^3^Department of Experimental Limnology, Leibniz-Institute for Freshwater Ecology and Inland Fisheries, Stechlin, Germany; ^4^Berlin-Brandenburg Institute of Advanced Biodiversity Research (BBIB), Berlin, Germany

**Keywords:** freshwater, lake periphyton, microbial interactions, Black Queen Hypothesis, OMICs tools

## Abstract

Microbial interactions play an essential role in aquatic ecosystems and are of the great interest for both marine and freshwater ecologists. Recent development of new technologies and methods allowed to reveal many functional mechanisms and create new concepts. Yet, many fundamental aspects of microbial interactions have been almost exclusively studied for marine pelagic and benthic ecosystems. These studies resulted in a formulation of the Black Queen Hypothesis, a development of the phycosphere concept for pelagic communities, and a realization of microbial communication as a key mechanism for microbial interactions. In freshwater ecosystems, especially for periphyton communities, studies focus mainly on physiology, biodiversity, biological indication, and assessment, but the many aspects of microbial interactions are neglected to a large extent. Since periphyton plays a great role for aquatic nutrient cycling, provides the basis for water purification, and can be regarded as a hotspot of microbial biodiversity, we highlight that more in-depth studies on microbial interactions in periphyton are needed to improve our understanding on functioning of freshwater ecosystems. In this paper we first present an overview on recent concepts (e.g., the “Black Queen Hypothesis”) derived from state-of-the-art OMICS methods including metagenomics, metatranscriptomics, and metabolomics. We then point to the avenues how these methods can be applied for future studies on biodiversity and the ecological role of freshwater periphyton, a yet largely neglected component of many freshwater ecosystems.

## Introduction

The term *periphyton* has a long history and describes a wide range of organisms living on various submerged substrates. Perhaps the most used and popular definition of periphyton communities was formulated by Wetzel (1983): “Periphyton is a complex community of microbiota (algae, bacteria, fungi, animals, inorganic, and organic detritus) that is attached to substrata. The substrata can be inorganic or organic, living or dead.” Similarly, at present, the term “periphyton” is applied to all forms of organismic structures (except macrophytes), which are growing on submerged materials, whereby the main emphasis is set on the algal component (Håkanson and Boulion, 2002; Vadeboncoeur et al., 2006). In microbial ecology the term *biofilm* is widely used and means microbial attachment to any kind of substrate mediated by an extracellular matrix. In aquatic ecosystems, biofilms include a wide variety of microbial organisms including microalgae (Donlan, 2002). Thereby, the term *periphyton* also includes macroscopic organisms and can be used more widely. In this paper, we provide an overview on studies performed on microbial organisms attached to any kind of substrate in aquatic ecosystems, and thus will use the term *periphyton* for the community of micro- and macroorganisms attached to submerged surfaces (with emphasis on microalgae and their associated bacteria) as well as the term *biofilm* for the whole array of microorganisms (e.g., Bacteria, Protista, fungi, and microalgae, etc.). In cases where the authors of the reviewed papers have used term *biofilm* synonymously for the attached community of auto- and heterotrophic microorganisms, we also use this term in the context of our above given definition.

Periphyton research has a long history and resulted in numerous biodiversity studies on (i) periphyton and its application as a tool for biological indication and assessment (Schneider and Lindsrom, 2011; Vadeboncoeur et al., 2011; Kelly, 2013; Cantonati and Lowe, 2014; DeNicola and Kelly, 2014), (ii) the role of periphytic communities in primary production (Winberg, 1960, 1980; Hill et al., 1992; Håkanson and Boulion, 2002; Vadeboncoeur and Steinman, 2002; Vadeboncoeur et al., 2011, 2014), (iii) stoichiometry (Hillebrand and Sommer, 1999; Hillebrand and Kahlert, 2001; Fitter and Hillebrand, 2009), and (iv) nutrient cycling (Toet et al., 2003; Teissier et al., 2007; Kalscheur et al., 2012). Some researches had noted a cultural and ecological importance of periphyton communities and regard periphyton in multiple ways, e.g., as solar-powered biogeochemical reactors, biogenic habitats, hydraulic roughness elements, early warning systems for environmental degradation, and troves of biodiversity (Larned, 2010). Therefore, periphyton is widely used as a model object for studies on effects of abiotic factors (physical disturbances, limiting nutrients, etc.), competitive interactions, and the significance of periphyton communities in carbon and nutrient cycling in aquatic food webs (e.g., Hansson, 1992; Hillebrand and Sommer, 1999; Holomuzki et al., 2010; Larned, 2010; Mulholland and Webster, 2010). An awareness of periphytic algal communities had increased after the approval of the EU Water Framework Directive (WFD; European Union, 2000). However, the most studies on freshwater periphyton have been carried out in streams and much less has been done in lakes (Cantonati and Lowe, 2014; DeNicola and Kelly, 2014; Kahlert and Gottshalk, 2014).

Analyses of biodiversity in large lakes have shown that the majority of species, including many endemic ones, occur in the littoral and profundal zones. This pattern is mainly caused by the high substrate heterogeneity, i.e., its physical complexity provides numerous niches that allow for co-existence of a high number of species. Thus, periphyton communities contribute considerably to biodiversity in freshwater ecosystems (Vadeboncoeur et al., 2011). Recent studies have shown that, particularly in shallow lakes, periphytic primary production is relatively high (Håkanson and Boulion, 2002; Vadeboncoeur et al., 2006, 2011, 2014; Brothers et al., 2013) and in some lakes it may even contribute to more than 80–90% of the total primary production (Wyatt et al., 2014; Vesterinen et al., 2016). Besides its considerable role for primary productivity, periphyton biofilms are also “hot-spots” for numerous biogeochemical processes, including denitrification, and thus play a crucial role in nutrient remineralization and element cycling (Toet et al., 2003; Teissier et al., 2007; Kalscheur et al., 2012).

Since recently there is an increasing interest in studies on the ecological role of periphytic communities, which is related to the use of periphyton for water purification due to its binding of toxic substances (Yang et al., 2016) as well as interactions with phytoplankton, macrophytes and macrobenthos (Do Nascimento Filho et al., 2019). Absolutely new direction in biofilm studies is colonization and degradation of microplastics (Kesy et al., 2019; Amaral-Zettler et al., 2020).

Numerous papers have been published in regard to the coupling between auto- and heterotrophs in periphyton communities (Hepinstall and Fuller, 1994; Rier and Stevenson, 2002; Scott et al., 2008; Rusanov et al., 2009). These studies revealed that microbial coupling depends to a large extent on environmental variables, such as nutrient fluxes (Tank and Dodds, 2003; Carr et al., 2005; Rusanov et al., 2009), light intensity (Roeselers et al., 2007), and availability of dissolved organic carbon (DOC; Sobczak, 1996; Rier and Stevenson, 2002), etc. During the past decades some physiological aspects of interactions between auto- and heterotrophs in freshwater periphytic biofilms have been studied (Bruckner et al., 2008, 2011; Allen et al., 2015), however, many mechanistic aspects of interactions in freshwater periphyton communities are still unknown and need to be examined in more details (Allen et al., 2015).

Recently, relevant protocols describing peculiarities of material collection, preservation, and extraction for molecular studies have been developed and assessed for freshwater habitats (Zimmermann et al., 2011; Paulsen and Holmes, 2014). Yet, despite the relatively high number of biodiversity studies on freshwater periphyton, molecular data allowing for the development of the new mechanistic concepts for studying function and hence ecological role of periphyton are still missing (Tsementzi et al., 2014; Pascault et al., 2015; Lindemann et al., 2017). Some studies on microbial interactions in marine planktonic and benthic communities allowed to develop several new fundamental concepts, e.g., about chemical signaling, quorum sensing, and inter-kingdom communication (Waters and Bassler, 2005; Grossart, 2010; Amin et al., 2015; Zhou et al., 2016), the Black Queen Hypothesis (Morris et al., 2012), phycosphere (e.g., Seymour et al., 2017), microbial cross-feeding (Smith et al., 2019), and other aspects.

**FIGURE 1:**
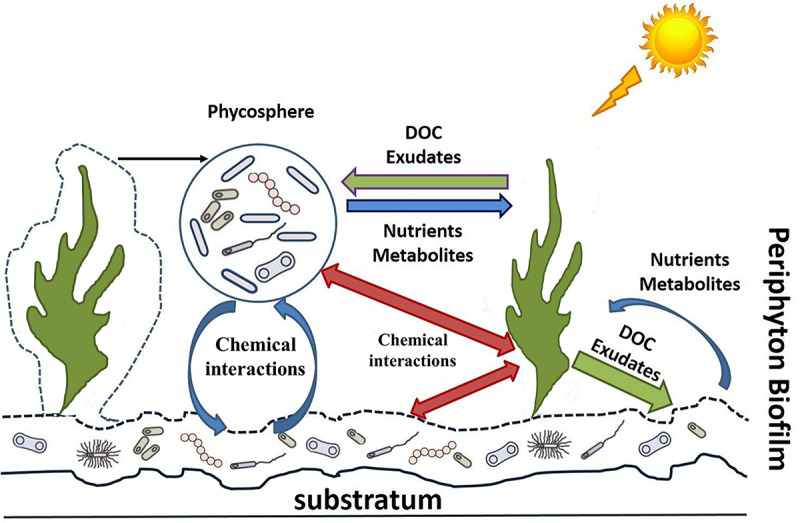
Microbial interactions in periphyton.

Our intention is to highlight the most recent concepts in microbial ecology with reference to the data obtained for marine and freshwater planktonic and benthic communities. We set a particular focus on microbial interactions to increase our understanding on the functional role of periphyton in lakes. A key question may arise, i.e., whether it is suitable to talk about freshwater periphyton when relying on data from other aquatic communities? In this paper we mainly focus on the role of algal-bacterial interactions in periphytic communities. It is well known that some algal phyla (i.e., Diatoms) can colonize the majority of aquatic habitats. Studies on algal-bacterial associations from different habitats have shown a major similarities in interactions between alga and bacteria, e.g., mutual control and selection of bacterial strains (Grossart et al., 2005; Burke et al., 2011a, b; Krohn-Molt et al., 2017; Stock et al., 2019). Since there are not many studies conducted on periphythic communities, we suppose that results and hypotheses obtained from other habitats can give us future directions for periphyton studies. We believe that application of new molecular methods—as has been done in other fields of aquatic ecology—will open up a plethora of new perspectives to studying numerous fundamental mechanisms of microbial activity and interactions in freshwater periphyton. For better guidance, in Figure 1 and Table 1 we have summarized the most prominent interactions and processes in freshwater periphyton and suggest several state of the art methods for their in-depth investigation.

**TABLE 1:** Overview of methods which can be used for investigation of major functional periphyton attributes.

**Type of interacting organism**	**Function**	**Methods of investigation**	**Achievements**
Autotrophs	DOC excretion	Wet oxidation, High temperature combustion, etc.	Data for ecosystem modeling
	Synthesis and excretion of exudates	Various biochemical methods, Metatranscriptomic and metabolomic analyses, Co-culturing experiments	Functional response, Metabolic interconnectedness
	Metabolic reactions	Combination of various OMICs tools	Functional response, Metabolic interconnectedness
	Chemical signaling and response	Number of chemical and biochemical methods, Combination of OMICs tools, Co-culturing experiments	Intra- and interspecies interactions
	Uptake of nutrients and bacterial metabolites (e.g., vitamins)	Metatranscriptomic and metabolomic analysis	Niche partioning, auxotrophy, Metabolic interconnectedness with bacteria
	Response on bacterial exudates and signaling molecules	Metatranscriptomic and metabolomic analysis	Alga-bacteria interactions
Heterotrophs	Loss-of-function mutations	Metagenomic and metatranscriptomic analyses	Adaptive gene loss, Cross-feeding
	Chemical signaling and response	Combination of OMICs methods, Co-culturing experiments	Alga-bacteria interactions, Interspecific interactions
	Metabolic reactions	Combination of OMICs methods	Cross-feeding, Interspecies interactions
	Biogeochemical reactions	Metatranscriptomic analysis, Various chemical and biochemical methods	Importance of community in biogeochemical cycles
	Response on autotrophic exudates and metabolites	Co-culturing experiments, Combination of OMICs methods	Algal-bacteria interactions

## Interactions Between Auto- and Heterotrophs in Aquatic Microbial Communities and Concepts Proposed

Principles and mechanisms of microbial interactions have been studied intensively for planktonic communities and cultures. Based on recent studies on microbial interactions in plankton, Grossart (2010) proposed the concept of an intertwined microbial network between free-living and surface-attached (particle-associated) bacteria, where surface associated bacteria may play a considerable role for nutrient cycling and biomass production. Later studies of free-living and particle-associated bacteria revealed that the connectivity between these communities increased along nutrient gradients (Hu et al., 2020). Also, it was shown that interactions in particle-associated community are stronger, than in free-living (Zhang et al., 2016; Hu et al., 2020). Microbial interactions mediated by chemical substances, e.g., allelopathy, seem to be a widespread strategy in both planktonic and benthic communities (Leflaive and Ten-Hage, 2007, 2009). So far, this concept has been frequently studied, in particular for interactions of pelagic bacteria with other organisms in pelagic environments, yet, only some studies aimed to decipher the connection of autotrophic periphyton with pelagic communities (Do Nascimento Filho et al., 2019; Dos Santos et al., 2020).

Unlike phytoplankton, biofilms represent specific organismic communities embedded in an extracellular polysaccharide matrix providing a favorable environment for short-distance microbial interactions (Jüttner, 1999; Battin et al., 2007). Studies on microbial biofilms and the main ecological concepts have been previously reviewed by Battin et al. (2007) indicating that biofilms should be viewed as complex microbial landscapes representing interconnected components of the larger landscape they colonize. Battin et al. (2007) mainly addressed the bacterial component of microbial biofilms. Later, in stream ecosystems, biofilms have been called “jungles” providing numerous ecological niches for a high number of organisms such as algae, protozoa, nematodes and even some insects, which are “among the top consumers in the stream biofilms” (Battin et al., 2016). At the same time, both local environment and biotic interactions act as drivers for local community composition (Rendueles and Ghigo, 2015). We suppose that aside of various close interactions between microbial organisms in biofilms, periphyton communities form an intertwined network with free-living bacteria (Grossart, 2010) and dead organic matter (Rusanov et al., 2009) in the surrounding environment.

Initially, in aquatic ecology, the “phycosphere” concept includes interactions between auto- and heterotrophic microorganisms, analogous to the rhizosphere concept in soils. Thereby, the phycosphere depicts an immediate region surrounding an algal cell, chain, or colony and represents a zone “in which bacterial growth is stimulated by the extracellular products of the alga” (Bell and Mitchell, 1972). Later studies on plankton confirmed that phytoplankton is surrounded by a diffusive boundary layer, in which excreted phytoplankton molecules accumulate in concentrations, high enough to be detected by bacteria allowing for intense communication and interaction with the algae (Karp-Boss et al., 1996; Jonsson et al., 2009; Amin et al., 2012). In the review of Ramanan et al. (2016), the phycosphere is compared with “an oasis for heterotrophic bacteria, constituting high concentrations of excreted organic carbon in the vast oligotrophic surroundings of both the ocean and inland waters.” At the same time, the authors point out that the phycosphere has been greatly neglected as a distinctive habitat for bacteria (Ramanan et al., 2015, 2016). Extending the phycosphere concept to the algal component of periphyton and taking into account that each species in these communities is in close contact with each other, the question arises: Do we regard the phycosphere of periphytic algae as a separate, specific ecological habitat occupied by distinct heterotrophic microbial communities? According to the concept proposed by Grossart (2010) this microbial community is interconnected by chemical means with its own members and with the surrounding periphytic and planktonic communities. Moreover, since in periphyton all organisms are in the most intimate contact, it leads to tight interactions among them. There are two main types of interactions: competitive and cooperative (Freilich et al., 2011). The cooperative concept lately was developed via the Black Queen Hypothesis, which proposes that cooperation can be a result of coevolution of coexisting bacteria and adaptive gene loss (Morris et al., 2012). This concept is discussed in more detail below.

Analysis of bacterial strains, isolated from more than 42 micro- and macroalgal species from marine and freshwater environments, revealed that bacterial species and strains having similar metabolic functions colonize similar algal taxa or groups (Burke et al., 2011a, b; Goecke et al., 2013). These metabolic functions imply the ability to degrade the complex polymers (such as polysaccharides), avoid competition, and to form mutualistic relationships with algae (Ramanan et al., 2015). In turn, this requires sharing the metabolic potentials of the interacting organisms and a complex signaling system (Ramanan et al., 2016).

Taking into account both the specificity of bacterial strains and the ability of algae to synthetize a high spectrum of active substances (Bazes et al., 2006; Bruckner et al., 2011), we may propose that algae controlling their phycosphere and attributed heterotrophic communities may represent key species or group of species defining the fate and function of microbial periphyton communities. In turn, bacterial communities influence many vital functions and the growth of algal hosts (Amin et al., 2012, 2015). Recent studies on diatom *Cylindrotheca closterium* and bacterial strains have shown a high specificity of bacteria and their algal hosts. Moreover, co-cultivation of this diatom with bacterial strains from a “foreign” algal host showed effects ranging from neutralism to antagonism with suppressing of algal growth (Stock et al., 2019). These results confirm the high specificity between algal host and its associated bacteria and suggest that algae can function as key-species for their associated bacterial communities.

Until recently, the methods for studying periphyton have not allowed us to dig into functional responses and interactions among individual members of periphyton communities. However, latest applications of metagenomics and metatranscriptomics together with metabolic reconstructions revealed many details of microbial interactions and their functional responses (Lindemann et al., 2017).

For example, synthesis of allelopathic substances, e.g., antibiotics and other inhibitors, by members of the biofilm community plays an essential role in determining microbial diversity and structure (Lenski and Riley, 2002; Burmolle et al., 2006). Studies on biofilms of different origin and bacterial associations with eukaryotic hosts, e.g., human pathogens (Harriott and Noverr, 2009; Lopes et al., 2012; Korgaonkar et al., 2013), as well as aquatic microbial and epiphytic communities (Burmolle et al., 2006; Allen et al., 2015; Kouzuma and Watanabe, 2015; Dittami et al., 2016), have revealed both synergistic and antagonistic inter-species interactions. It is well-known that bacteria are able to stimulate growth, morphogenesis, and zoosporogenesis of algae (Marshall et al., 2006; Goecke et al., 2013) as well as the synthesis of algal extracellular compounds (Bruckner et al., 2011). Bacteria serve as an ultimate source of cobalamin for vitamin B dependent algae (Cole, 1982; Kazamia et al., 2012) and promote algal growth via excretion of substances with hormonal nature (De-Bashan et al., 2008). In turn, DOC, excreted by algae serve as nutrients for alga-associated heterotrophic microbial community (Kazamia et al., 2012; Wyatt et al., 2014). Associations with bacteria can greatly increase the ability of algae to survive in unfavorable conditions. For example, studies on cell cultures of the brown alga *Ectocarpus* had shown that presence of specific associated bacteria increase the tolerance of the algal host to low salinity (Dittami et al., 2016). Besides synergistic interactions, a multispecies community inevitably faces competitive antagonistic interactions among species (Freilich et al., 2011; Allen et al., 2015; Yuan and Meng, 2020). Last, studies have shown that in the absence of carbon limitation, heterotrophs can out-compete algae for nutrients and thus suppress algal growth (Wyatt et al., 2019). Studies on freshwater periphyton revealed a frequent presence of allelopathic compounds within microbial biofilms (Jüttner and Wu, 2000; Leflaive et al., 2008) and the capability of many benthic species to produce various inhibiting substances with a highly variable chemical nature (Hagmann and Jüttner, 1996; Hirata et al., 2003; Pouvreau et al., 2007; Vanelslander et al., 2012). These substances include antibiotics and enzymes, which can be transported to competitors with extracellular vesicles and affect cellular differentiation, protein synthesis, and inhibit cell-to-cell signaling (Stubbendieck and Straight, 2016). Studies on macro- and microalgae indicate that several specific algal compounds such as fatty acids including α-linolenic (18:3 n–3) and eicosapentaenoic acids (22:5 n–3) have a strong antibacterial activity as well (Debois et al., 2010; Smith et al., 2010; Allen et al., 2015). Inhibiting the growth of both bacteria and other algae may control and shape the associated microbial communities (Allen et al., 2015).

Thus it is not surprising that studies on various microbial biofilms reveal that cell-to-cell chemical signaling plays a crucial role in a shaping of the biofilm community, e.g., by controlling processes such as invasion and colonization (Ben Jacob et al., 2004; Burmolle et al., 2006; Battin et al., 2007). Since many signaling molecules are small, they can diffuse freely through the highly hydrated biofilm matrix, depending on their hydrophobicity (Lovett et al., 2005). However, the extracellular polymeric substances have a high chemical heterogeneity creating environmental gradients which may serve as signal filters (Neu et al., 2001) or fluid channels (Espinosa-Urgel, 2003). At the same time, flow of water may dilute the pool of signaling molecules or transfer them to the more distant biofilm areas (Battin et al., 2007) resulting in chemical interactions with the surrounding environment.

Enriched cultures of planktonic bacterial communities (Garcia et al., 2015) have been studied via metatranscriptome analysis and revealed the many details of chemical interactions which may also occur in periphyton communities. However, peculiarities of periphytic communities lead to some technical difficulties. The tight relationship between bacteria and algal host (Koedooder et al., 2019), for example, render it difficult to obtain axenic cultures. Although the data on associated bacteria of green microalgae have shown that only a few species of bacteria have tight relationship with their algal host (Krohn-Molt et al., 2017), the high functional interconnectedness in periphytic communities likely result in the presence of a high number of associated, yet, non-culturable bacteria. This may result in methodical difficulties in selecting the right experimental bacterial strains, experimental design, and correct data processing. A possible way to solve these difficulties could be the joint usage of metagenomics, single cell genomics, when single the cell is isolated from the algal community together with extended microbiome and metatranscriptomic analyses (Krohn-Molt et al., 2013, 2017). Since recently, data sets of metagenomes of natural populations (i.e., biofilms) have increased significantly (Feio et al., 2020) facilitating improved data processing.

Yet, fundamental aspects of inter-species interactions among bacteria as well as among pro- and eukaryotic organisms in periphyton are still poorly understood, and their studies require the use of new methods and concepts including state-of-the-art OMICs approaches, specifically designed for complex and intertwined periphyton communities.

## “Black Queen Hypothesis”: Can We Apply it to Freshwater Periphyton?

Recent applications of new molecular methods for studies on bacterial interactions of free-living lineages of oceanic plankton have resulted in the proposition of the so-called “Black Queen Hypothesis” (BQH). It suggests that some species of free-living bacteria are dependent on functions of co-occurring bacteria due to loss of a number of genes during reductive genome evolution. The hypothesis of cross-feeding assigns co-existing species as “helpers” and “beneficiaries.” The scenarios of co-existence in microbial communities are diverse and include a number of strategies (Elias and Banin, 2012). Recently bacterial cross-feeding assigned to 4 different types, depending on the strategy of interacting bacteria (Smith et al., 2019). They include interactions between self-sufficient organisms as well as auxotrophy, with and without gene loss. Thereby, the interacting strains cross-feed on metabolites of each other and gain benefit directly or indirectly providing benefit via reduction of the required energy costs (Smith et al., 2019).

Unlike many theories of coevolution, e.g., the Red Queen Hypothesis (Van Valen, 1973), the BQH proposes that relationships between helpers and beneficiaries doesn’t necessarily arise from direct interactions, but beneficiaries can simply stop a costly function that is provided by their helpers (loss-of-function mutation; Morris et al., 2012). This new paradigm caused an intense discussion in the scientific community and suggests that bacteria often form interdependent cooperative interactions in communities and develop a clear genomic signature via complementary in loss of shared diffusible functions (Sachs and Hollowell, 2012).

Metatranscriptomic studies on a mixed freshwater model culture, containing previously uncultured bacteria, have revealed a metabolic interconnectedness and niche partitioning between bacterial community members. It implies that every species in microbial communities may “play their own violin in a common concerto” by performing functions vital for the respective co-existing species (Garcia et al., 2015). Experiments with mixed algal-bacterial co-cultures have shown that in periphyton heterotrophic bacteria play a crucial ecological role and that algae-only assemblages develop slower and are less stable than in the presence of bacteria (Roeselers et al., 2007; Lubarsky et al., 2010; Kouzuma and Watanabe, 2015; Koedooder et al., 2019). Therefore, it is difficult to obtain representative axenic cultures of periphytic algae. This functional interconnectedness between photo- and heterotrophic consortia in algal-bacterial associations and, particularly, in periphyton is consistent with the above portrayed BQH (Morris et al., 2012). Yet, such interconnectedness raises the following questions: is this functional interrelationship obligatory and a result of (i) gene loss described by the BQH (when organism with lost-of-function mutation relies on self-sufficient organisms)? (ii) the proposed cross-feeding scenarios? or (iii) phenotypic plasticity caused by the present environmental conditions (Smith et al., 2019)?

Loss-of-function mutations are predicted to be the most widespread among intimately interacting bacteria, e.g., biofilm and rhizosphere consortia. Models suggest that within these communities deletions might often occur in a complementary fashion among various interacting bacteria (Morris et al., 2012; Sachs and Hollowell, 2012). Yuan and Meng (2020) modeled the development of biofilms in a bioreactor and provided an overview of prevailing interactions. They found a difference in interactions in mobile (flocks) vs. those in surface-attached biofilms. In flocks, the prevailing interactions were more competitive corresponding more with the Red Queen Hypothesis. In surface-attached biofilms, however, the relationships were less competitive and more cooperative. However, as the authors note themselves, their experiment had lasted for a too short time to demonstrate the ecological effects of possible loss-of-function mutations (Yuan and Meng, 2020).

One of the last stochastic-spatial models, proposed by Stump et al. (2018), shows that in free-living, rather instable systems self-sufficient species (which produce all required resources) have an advantage in comparison to non-producers or those who produce only one resource from the number of resources required. Spatial structure promotes an increase in mutualistic relationships vs. self-sufficiency which is in agreement with data from natural systems. According to this model, the neighbor uncertainty is an important factor increasing the cost of loss-of-functions mutations and gives more advantage to self-sufficiency vs. mutualism according to the BQH. However, the authors also note that their model doesn’t include chemical interactions between specimens, which take place in natural systems and may substantially decrease the effect of neighbor uncertainty. When taking into account chemical signaling and chemotactic motility, the advantage can be shifted more to the side of non-producers and co-existing one-producers (Stump et al., 2018). However, in the case of biofilms and algal-bacterial interactions, microbial communities face selection according to substrate type and ability to co-exist with a specific algal host and other microbes (Goecke et al., 2013; Stock et al., 2019; Yuan and Meng, 2020). This decreased effect of neighbor uncertainty can result in strengthening mutualistic relationships via the BQH. Also, studies on diatom-bacterial interactions reveal a high species-specific interdependence of the algal host and bacteria (Grossart et al., 2005; Stock et al., 2019). Moreover, bacteria can also influence the diatom community composition themselves by promoting growth of one species and suppressing growth of others (Koedooder et al., 2019).

Thus, communities of periphyton and diatom-bacteria have a great potential for detailed evaluation of mechanisms of cross-feeding and loss-of-function mutations.

## New Methods, New Concepts

For a long time, difficulties in methodology have greatly limited studies on microbial communities and algal-bacterial associations, in particular since more than 99% of bacteria from natural habitats still remain undetectable by traditional microbiological methods and are not amenable for cultivation (Curtis, 2002; Zarraonaindia et al., 2013; Solden et al., 2016). As a result, the majority of bacterial diversity has been missed in physiological studies for understanding the ecological role of heterotrophic bacteria in aquatic ecosystems (Pace, 1997). Implementation of molecular methods to ecology allowed to improve our knowledge on microbial communities, their diversity, succession, ecological functions, and physiological responses (Jackson et al., 2001; Fechner et al., 2010; Goecke et al., 2013). During the past two decades, OMICs methods have been developed and successfully applied in aquatic ecology research. Metagenomics allows to retrieve (almost complete) genome sequences from complex microbial assemblages by avoiding cultivation biases (Moran, 2009) and thus provides a useful method to address the functional potential of a given periphyton organism. However, with periphyton and other aquatic biofilms the use of molecular methods meets some difficulties. First, periphytic communities are very heterogeneous often resulting in very variable results. Second, due to the presence of extracellular DNA as well as dead and inactive cells, DNA extracted from the sample may result in an overestimation of the true diversity (Stal et al., 2019). Another way to obtain DNA from environmental samples is single cell genomics, i.e., genomic DNA is extracted from a single, mechanically isolated cell. However due to biases in amplification, it results incomplete genomes. Thus, the best result can be achieved with the joint use of these complimentary methods (Solden et al., 2016). In addition, these methods can be complemented with metaproteomics and metabolomics. Metaproteomics can be defined as “the large-scale characterization of the entire protein content of the environmental microbiota at a given point in time” (Wilmes and Bond, 2004) aiming to uncover the response of any community through expressed proteins, protein paths, and networks (Courant et al., 2014; Wilmes et al., 2015). In contrast, metabolomics investigates the complete pool of low molecular weight compounds (metabolites) in microbial communities and their changes in response to external stressors or interactions (Lankadurai et al., 2013). Both methods can be also utilized for quantification.

In contrast, metatranscriptomics reveals the genes expressed at specific environmental conditions at the moment of sampling and thus hint on specific physiological functions (Moran, 2009; Moran et al., 2013). Metatranscriptomics represents an OMICs method, which allows to disentangle the specific response of microbial species to different environmental factors including organismic interactions. As recently shown for pelagic systems, this methodology opens new perspectives in studies on interactions between algae and bacteria in freshwater periphyton, especially because it reveals the specific response of individual species at the level of gene products. Consequently, metatranscriptomics deals with a subset of genes responsible for important functions, such as nutrient metabolism and organismic interactions (Warnecke and Hess, 2009; Alexander et al., 2015). Most of such metatranscriptomic studies, however, have been conducted with plankton communities, mainly in marine and coastal environments (Krohn-Molt et al., 2013, 2017; Alexander et al., 2015; Amin et al., 2015; Garcia et al., 2015). In the case of benthic communities this method meets some challenges. In environmental samples, transcripts are low in abundance relative to the total rRNA pool. At the same time these samples (and periphytic communities as well) are rich of inhibiting compounds. These obstacles, however, can be overcome by rigorous extraction to remove RNAase enzymes, use of polyA-enrichment (if Eukaryota are targeted), and removing the rRNA with special reagents (Kuske et al., 2015). Only recently, relevant protocols became available (Zimmermann et al., 2011; Krohn-Molt et al., 2013, 2017; Paulsen and Holmes, 2014; Hovde et al., 2015), which can also be applied for freshwater periphyton. We thus believe that the application of metatranscriptomics in combination with metagenomics and single-cell genomics, which can provide the evidence of gene loss, as well as other OMICs approaches are suitable for periphyton studies and capable to reveal the basic mechanisms of chemical signaling and functional interconnectedness, both are crucial for co-existence and interactions of periphytic communities.

## Conclusion

Periphyton and its microbial interactions (Figure 1) within a diverse microbial community are of great importance for whole lake primary production, nutrient cycling, and food-web structure, particularly in shallow lakes. Development and application of molecular methods in aquatic microbial ecology provide new perspectives revealing microbial diversity and the underlying mechanisms of alga-bacteria interactions, which are crucial for a mechanistic understanding of the specific ecological role of freshwater periphyton. Metatranscriptomic analysis, combined with co-culturing experiments (when a few species are cultivated together), holds the promise to reveal the mechanisms of cell-to-cell communication, signaling, niche portioning, and functional response of every interacting species in the community (Table 1). Metatranscriptome analysis together with metagenomics and other OMIC’s methods corroborated the concepts of chemical signaling in algal-bacterial interactions (Amin et al., 2015) and resulted in the proposition of the Black Queen Hypothesis in pelagic or marine benthic systems. Since in periphyton all organisms are in close interaction, these communities are very promising for further studies on species co-evolution, e.g., in the view of various hypotheses such as the Red vs. the Black Queen Hypotheses. Recent findings allow to complement and expand existing concepts of freshwater periphyton communities as interconnected microbial networks inside of diverse microbial landscapes. Development of the new concepts, based on microbial genome evolution and chemical signaling, are promising to improve our knowledge on microbial interactions and their ecological role, in particular in freshwater periphyton communities. Combination of metagenomic and metatranscriptomic techniques with more quantitative tools such as metaproteomics and metabolomics is required to uncover the ways and mechanisms of microbial interactions. In turn, a better understanding of these mechanisms enables us to predict better the ecological consequences of environmental changes (natural or man-made). The knowledge of these mechanisms in lake

periphyton is crucial for effective management and conservation of freshwater resources in a changing world.

## Author Contributions

YG analyzed the literature, concepts, wrote large parts of the text, and discussed it with the coauthor. H-PG initiated the view on periphyton from the point of microbial interactions and application of the newest concepts and methods, discussed and edited the manuscript. Both authors revised the manuscript. Both authors contributed to the article and approved the submitted version.

## Conflict of Interest

The authors declare that the research was conducted in the absence of any commercial or financial relationships that could be construed as a potential conflict of interest.
